# Mitochondrial DNA Polymorphisms of Peripheral Blood Mononuclear Cells Associated with Sustained Ventricular Tachycardia in Patients with Cardioverter-Defibrillator Implantation Indications

**DOI:** 10.31083/RCM26744

**Published:** 2025-03-17

**Authors:** Tariel Atabekov, Viacheslav Korepanov, Sergey Krivolapov, Mikhail Khlynin, Sergey Afanasiev, Maria Golubenko, Roman Batalov, Sergey Popov

**Affiliations:** ^1^Department of Surgical Arrhythmology and Cardiac Pacing, Cardiology Research Institute, Tomsk National Research Medical Center, Russian Academy of Sciences, 634012 Tomsk, Russia; ^2^Laboratory of Molecular and Cellular Pathology and Gene Diagnostics, Cardiology Research Institute, Tomsk National Research Medical Center, Russian Academy of Sciences, 634012 Tomsk, Russia; ^3^Laboratory for Population Genetics, Research Institute of Medical Genetics, Tomsk National Research Medical Center, Russian Academy of Sciences, 634050 Tomsk, Russia

**Keywords:** sustained ventricular tachycardia, mitochondrial DNA, peripheral blood mononuclear cells, cardioverter-defibrillator

## Abstract

**Background:**

Mitochondrial dysfunction in myocardium cells has been implicated in arrhythmogenesis, including ventricular tachycardia (VT). A carriage of point mitochondrial DNA (mtDNA) polymorphisms may contribute to the risk of certain arrhythmias. Therefore, it is hypothesized that mtDNA genotype could predict the risk of sustained VT (_S_VT). We aimed to explore whether specific mtDNA polymorphisms of peripheral blood mononuclear cells (PBMC) can serve as biomarkers for predicting the risk of _S_VT in patients with indications for an implantable cardioverter-defibrillator (ICD).

**Methods:**

A total of 122 patients with ICD implantation indications who underwent transthoracic echocardiography (TTE) were enrolled in the study. Total DNA from PBMC was isolated using the phenol-chloroform extraction method. Genotyping of mtDNA polymorphisms A2706G, G3010A and G9055A was performed using restriction fragment length polymorphism analysis. Correlations between clinical parameters and mtDNA polymorphisms with _S_VT registered prior to ICD implantation were evaluated. Based on our data, we developed a risk model for _S_VT.

**Results:**

Prior to ICD implantation, 70 (56.6%) patients had _S_VT (1st group) and 52 (43.4%) patients did not have _S_VT (2nd group). Patients with _S_VT were significantly older than patients without _S_VT (66.9 ± 9.9 year vs. 59.5 ± 10.6 year, *p* < 0.001), had a lower value estimated glomerular filtration rate (_e_GFR) (65.7 ± 19.7 mL/min/1.73 m^2^ vs. 77.9 ± 16.1 mL/min/1.73 m^2^, *p* < 0.001) and less frequently had A2706G mtDNA polymorphism (55.7% vs. 76.9%, *p* = 0.015). According to the multivariable logistic regression, age (odds ratio (OR) = 1.055, 95% confidence interval (CI) 1.009–1.103, *p* = 0.017), _e_GFR (OR = 0.974, 95% CI 0.949–0.999, *p* = 0.041) and absence of A2706G mtDNA polymorphism (OR = 0.335, 95% CI 0.141–0.797, *p* = 0.013) were independently associated with the _S_VT. We constructed a logistic equation with calculation of the cut-off value. The discriminative ability of the receiver operating characteristic curve (area under the curve) was 0.761 (95% confidence interval 0.675–0.833; sensitivity 65.71%; specificity 76.92%).

**Conclusions:**

In patients with ICD implantation indications, a carriage of mtDNA polymorphism A2706G is associated with _S_VT. Our risk model including age, _e_GFR and absence of A2706G mtDNA substitution was able to distinguish patients with _S_VT. Further investigations of their predictive significance are warranted.

**Clinical Trial Registration:**

NCT03667989 (https://clinicaltrials.gov/study/NCT03667989).

## 1. Introduction

The major cause of death worldwide is sudden cardiac death (SCD), with an estimated United States and Russian Federation annual incidence of 350 thousand and 142–473 thousand, respectively [1, 2]. Most of these deaths are associated with life-threatening ventricular tachyarrhythmias (VTA) such as sustained ventricular tachycardia (_S_VT) and ventricular fibrillation (VF). Implantable cardioverter-defibrillators (ICD) are the primary management strategy for VTA and they have been proven to reduce mortality rates [3]. However, this type of SCD prevention is essentially a palliative method and there is currently no effective preventive treatment strategy, because the mechanisms underlying fatal arrhythmias are still poorly understood and risk stratification of VTA remains a major unsolved clinical problem.

Dysfunction of mitochondria may contribute to a wide range of cardiovascular pathologies including arrhythmias [4]. Mitochondrial dysfunction of myocardium cells is implicated in VTA arrhythmogenesis, and plays a crucial role in their development [5]. Mitochondrial DNA (mtDNA) polymorphism, in turn, can influence the function of the encoded subunits of respiratory chain complexes [6, 7]. Based on the data obtained from patients with atrial fibrillation, it was revealed that carriage of haplogroup H probably has a protective effect regarding the development of persistent atrial fibrillation compared with haplogroup U [8]. It has also been shown that a decrease in the mtDNA copy numbers is associated with the development of atrial fibrillation [9]. Studying mitochondrial dysfunction in myocardium cells is only possible after a biopsy or radionuclide assessment, which is fraught with its own difficulties and potential complications [10]. Peripheral blood mononuclear cells (PBMC) are a group of mononuclear cells that exist in blood and exhibit important roles in the body’s immune responses [11]. There are numerous mitochondria in each cell of the body, and each of them has up to 10 mtDNA copies [12]. In cardiovascular disease, the number of mtDNA will change to become comparable to an individual without the disease. Therefore, it could be hypothesized that mtDNA polymorphisms might contribute to the risk of _S_VT. The aims of our study were to evaluate the association of mtDNA polymorphisms with _S_VT in patients with ICD implantation indications and explore whether specific mtDNA polymorphisms can serve as biomarkers for predicting the risk of _S_VT.

## 2. Materials and Methods

### 2.1 Patient Population 

Patients with ICD indications (secondary and primary prevention of SCD) were enrolled in this nonrandomized, single-center, clinical, open, cross-sectional observational study. Individuals with myocardial infarction (MI) less than 3 months old, hypertrophic cardiomyopathy, severe concomitant pathology (end-stage renal failure and hepatic insufficiency, cancer of any location), cognitive dysfunction and indications for revascularization, and young patients (<18 year) were not included in the study.

All patients underwent the full physical (6-minute walk distance test (6MWDT), transthoracic echocardiography (TTE), 12-lead electrocardiography (ECG), 24-hour Holter monitoring ECG, coronary angiography and blood analyses) and additional (mitochondrial nucleotide transition assessment using PBMC) examination. All patients received basic therapy in accordance with the guidelines for the management of ventricular tachycardia (VT) and heart failure (HF). All included patients were from a registered study (ClinicalTrials.gov, NCT03667989).

### 2.2 Transthoracic Echocardiography Acquisition and Analysis

The echocardiography was performed using the Philips HD15 PureWave ultrasound machine (Philips Ultrasound, Inc., Bothell, WA, USA). The TTE was carried out from standard positions with the intracardiac hemodynamic parameters assessment and determination of the dimensional, volume and indexed indicators of the heart chambers and left ventricular ejection fraction (LVEF). The right and left ventricles contractility and cardiac valve function, with the exception of the pulmonary valve, were evaluated.

### 2.3 DNA Isolation and Genotyping

Blood sampling was performed before ICD implantation. DNA extraction from the whole ethylenediaminetetraacetic acid (EDTA) blood was carried out by phenol chloroform extraction. Isolated DNA samples were placed for storage at –20 °C until further stages of the study. The DNA sample quality and concentration were evaluated using a NanoDrop – 2000C spectrophotometer (Thermo Fisher Scientific, Waltham, MA, USA).

Three mtDNA polymorphisms were selected for the analysis, namely A2706G, G3010A and G9055A. The positions are numbered according corrected reference human mtDNA sequence [13]. Genotyping of the polymorphisms was carried out by restriction analysis. For this aim, we performed polymerase chain reaction (PCR) analysis using specific primers for each polymorphism [14]. Primer annealing conditions for PCR were identified individually for each primer pair. Resulting amplicons then underwent restriction fragment length polymorphism analysis using site-specific endonucleases (SibEnzyme Ltd., Novosibirsk, Russia). The mixture for the restriction procedure included PCR-product (10 µL), the restriction buffer supplied with the enzyme (1.2 µL), 1 U of the enzyme, and deionized water to a final volume of 12 µL. The mixture was incubated for 12–24 hours at the temperatures optimal for each enzyme, according to the manufacturer’s information. Separation of restriction products by size was carried out immediately after incubation with 2% agarose gel electrophoresis for 40 min at a voltage of 130 V with staining with ethidium bromide. Visualization and confirmation of the obtained results were carried out in ultraviolet light on the Gel Doc documentation system (BioRad, Hercules, CA, USA).

### 2.4 Clinical Evaluation and Study Design

**Fig. 1.**
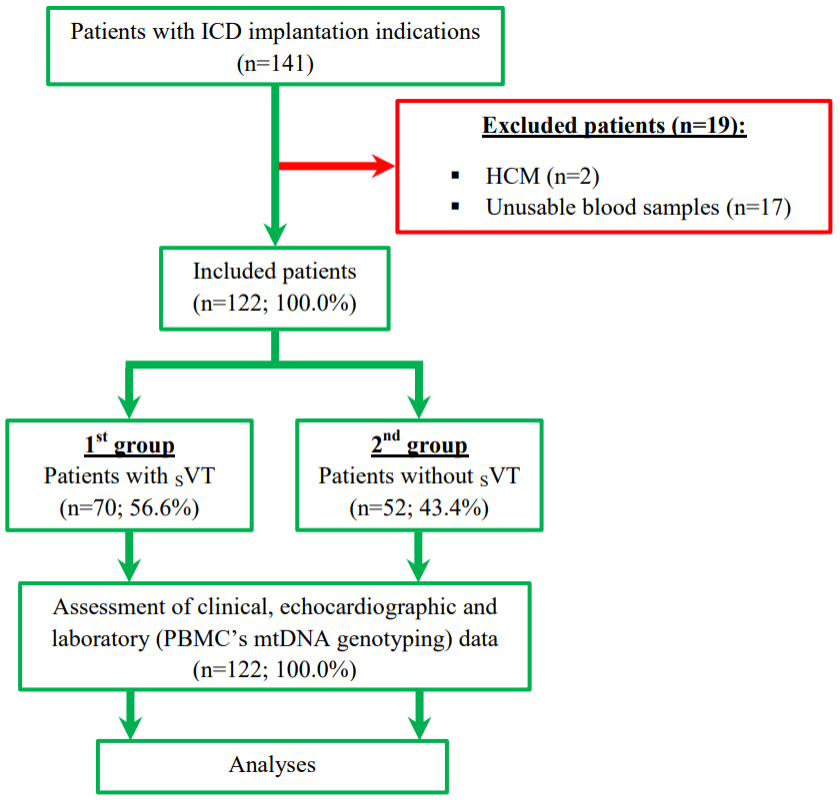
**Study flow chart and design**. Abbreviations: HCM, hypertrophic cardiomyopathy; ICD, implantable cardioverter-defibrillator; mtDNA, mitochondrial DNA; PBMC, peripheral blood mononuclear cells; _S_VT, sustained ventricular tachycardia.

Information on clinical assessment was obtained for all of the 122 patients included in the study. All participants were divided into 2 cohorts in accordance with the _S_VT presence. The _S_VT criteria were 3 or more consecutive ventricular beats with rate of 120 or more beats per minute and duration for 30 or more sec. The 1st group included patients with _S_VT, the 2nd group – without _S_VT. The flow chart and study design are presented in Fig. 1.

### 2.5 Statistical Methods

All qualitative and categorical variables are presented as counts (n) and percentages (%). The continuous variables are presented as the mean (M) ± standard deviation (SD) for normally distributed variables or as the median (Me) and interquartile ranges [Q1; Q3] for non-normally distributed variables. Kolmogorov-Smirnov, Lilliefors and Shapiro-Wilk tests were used to test the normality of continuous data distribution. Differences between groups for continuous data with normal distribution were assessed using the two-sided Student’s *t*-test. The Mann–Whitney U-test was used for independent ordinal or non-normally distributed data. The significance of differences between the groups was determined using the nonparametric Chi^2^ or Fisher’s exact test. A comparison between patients with and without sustained VT was performed for the complete dataset. Statistical analysis was performed with Statistica 10.0 (StatSoft Inc., Tulsa, OK, USA) and Medcalc 19.2.6 (MedCalc Software, Ostend, Belgium). Statistical significance was defined by a *p*-value < 0.05.

### 2.6 Risk Stratification Analysis and Predictive Model Development

The binary logistic regression analysis was used to distinguish variables associated with the _S_VT. The multicollinearity was excluded using the non-parametric Spearman analysis. Based on pairwise correlation coefficients, the presence of collinear factors was revealed. Factors were considered collinear if R >0.7. Only one of the interrelated variables was added to the model. The multivariable logistic regression analysis was performed using a stepwise elimination technique. Only indicators with *p* < 0.05 were added in the final model. Goodness-of-fit was performed by the Hosmer-Lemeshow test. The Pearson correlation and *t*-test were used to examine the significant correlations between the predictors. Regression analysis results were presented as odds ratios (ORs) with 95% confidence intervals (CIs). Odds ratios and the beta coefficients used in the logistic equation could be converted into each other: OR = eβ.

Finally, three variables (age, estimated glomerular filtration rate (_e_GFR) and absence of mtDNA A2706G transition), independently associated with _S_VT, were added to the predictive model. The area under the curve (AUC) was calculated to assess the discriminatory ability of the risk stratification score.

## 3. Results

### 3.1 Patient’s Demographic and Clinical Data

In 141 patients with an ICD implantation indication, we excluded 19 patients with failed blood samples (n = 17) and hypertrophic cardiomyopathy (n = 2). For the remaining 122 (100.0%) participants, the mean age was 63.7 ± 10.8 years and 94 (77.0%) patients were males. The 1st group included 70 (56.6%) patients with _S_VT. The 2nd group included 52 (43.4%) patients without _S_VT. The baseline characteristics of participants are presented in Table 1. Patients with _S_VT were significantly older than patients without _S_VT (66.9 ± 9.9 year vs. 59.5 ± 10.6 year, *p* < 0.001). The subgroups of patients also differed significantly by the portion of patients with I (7.1% vs. 25.0%, *p* = 0.006) and II (65.7% vs. 38.4%, *p* = 0.002) New York Heart Association (NYHA) class, but the 6MWDT indicator didn’t differ between groups (*p* = 0.635). Individuals with _S_VT were more likely to have ventricular fibrillation (8.6% vs. 0.0%, *p* = 0.030), lower _e_GFR (65.7 ± 19.7 mL/min/1.73 m^2^ vs. 77.9 ± 16.1 mL/min/1.73 m^2^, *p* < 0.001) and left ventricular end-systolic volume (74.5 [42.0; 106.0] mL vs. 147.5 [114.0; 205.0] mL, *p* < 0.001), and higher LVEF (47.5 [41.0; 62.0]% vs. 32.5 [28.0; 36.0]%, *p* < 0.001). Significant differences in echocardiographic indicators were due to the fact that all patients without _S_VT had primary SCD prevention indications for ICD implantation. Also, patients without _S_VT were more likely to have prescribed loop diuretics (59.6% vs. 27.1%, *p* < 0.001), mineralocorticoid receptor antagonists (84.6% vs. 50.0%, *p* < 0.001), angiotensin receptor/neprilysin inhibitors (34.6% vs. 8.6%, *p* < 0.001) and sodium glucose co-transporter 2 inhibitors (44.2% vs. 10.0%, *p* < 0.001) due to the fact that patients have HF with reduced LVEF and received the therapy in accordance with the current guidelines for the HF management. Patients with _S_VT were more likely to have longer baseline corrected QT interval (QTc) (429.3 ± 33.8 ms vs. 419.8 ± 31.3 ms, *p* = 0.031). This significant difference was due to the fact that the majority of patients with _S_VT were treated with amiodarone (62.8% vs. 38.4%, *p* = 0.007). Also, patients with _S_VT were more likely to have lipid-lowering treatment (92.8% vs. 80.7%, *p* = 0.044).

**Table 1.** **Baseline characteristics of the patients**.

Demographic and clinical characteristics		Overall population (n = 122)	1st group Pts with _S_VT (n = 70)	2nd group Pts without _S_VT (n = 52)	*p*_2-3_
		1	2	3	
Age, year, M ± SD		63.7 ± 10.8	66.9 ± 9.9	59.5 ± 10.6	<0.001
Male gender, n (%)		94 (77.0)	53 (75.7)	41 (78.8)	0.684
History of myocardial infarction, n (%)		73 (59.8)	45 (64.3)	28 (53.8)	0.244
History of coronary artery stenting, n (%)		48 (39.3)	28 (40.0)	20 (38.4)	0.863
History of CABG, n (%)		29 (23.8)	19 (27.1)	10 (19.2)	0.310
Baseline New York Heart Association class:					
	I, n (%)	18 (14.7)	5 (7.1)	13 (25.0)	0.006
	II, n (%)	66 (54.1)	46 (65.7)	20 (38.4)	0.002
	III, n (%)	38 (31.1)	19 (27.1)	19 (36.5)	0.267
	Baseline 6MWDT, m, M ± SD	353.9 ± 85.3	352.1 ± 77.3	356.3 ± 95.8	0.635
Arrhythmias prior to ICD implantation:					
	History of _S_VT, n (%)	70 (56.6)	70 (100.0)	0 (0.0)	<0.001
	Paroxysmal atrial fibrillation, n (%)	46 (37.7)	30 (42.8)	16 (30.7)	0.173
	Ventricular fibrillation, n (%)	6 (4.9)	6 (8.6)	0 (0.0)	0.030
Comorbidities:					
	Hypertension, n (%)	41 (33.6)	26 (37.1)	15 (28.8)	0.337
	Diabetes mellitus, n (%)	26 (21.3)	18 (25.7)	8 (15.4)	0.168
	Baseline body mass index, kg/m^2^, M ± SD	28.9 ± 5.1	29.2 ± 4.8	28.5 ± 5.5	0.324
	Dyslipidemia, n (%)	61 (50.0)	37 (52.8)	24 (46.1)	0.464
	Baseline _e_GFR, mL/min/1.73 m^2^, M ± SD	70.9 ± 19.2	65.7 ± 19.7	77.9 ± 16.1	<0.001
	Chronic obstructive pulmonary disease, n (%)	16 (13.1)	10 (14.3)	6 (11.5)	0.656
	Stroke, n (%)	3 (2.4)	1 (1.4)	2 (3.8)	0.393
Baseline electrocardiographic and echocardiographic findings:					
	Baseline QTc, ms, M ± SD	425.3 ± 33.0	429.3 ± 33.8	419.8 ± 31.3	0.031
	LV end-systolic volume, mL, Me (Q_1_; Q_3_)	105.0 (59.0; 150.0)	74.5 (42.0; 106.0)	147.5 (114.0; 205.0)	<0.001
	LV ejection fraction, %, Me (Q_1_; Q_3_)	40.0 (32.0; 55.0)	47.5 (41.0; 62.0)	32.5 (28.0; 36.0)	<0.001
Baseline therapy:					
	Beta-blockers, n (%)	110 (90.2)	61 (87.1)	49 (94.2)	0.193
	Loop diuretics, n (%)	50 (41.0)	19 (27.1)	31 (59.6)	<0.001
	Mineralocorticoid receptor antagonists, n (%)	79 (64.7)	35 (50.0)	44 (84.6)	<0.001
	ACEI, n (%)	72 (59.0)	44 (62.8)	28 (53.8)	0.316
	Antiplatelet agents, n (%)	72 (59.0)	41 (58.6)	31 (59.6)	0.907
	Lipid-lowering treatment, n (%)	107 (87.7)	65 (92.8)	42 (80.7)	0.044
	Angiotensin II receptor blocker, n (%)	24 (19.7)	18 (25.7)	6 (11.5)	0.051
	ARNi, n (%)	24 (19.7)	6 (8.6)	18 (34.6)	<0.001
	Amiodarone, n (%)	64 (52.4)	44 (62.8)	20 (38.4)	0.007
	Anticoagulants, n (%)	52 (42.6)	34 (48.6)	18 (34.6)	0.123
	Hypoglycemic drugs, n (%)	16 (13.1)	11 (15.7)	5 (9.6)	0.323
	SGLT2i, n (%)	30 (24.6)	7 (10.0)	23 (44.2)	<0.001

Values in bold indicate statistical significance. The values are signified as M ± SD and Me (Q_1_; Q_3_) for continuous and n (%) for categorical indicators. Abbreviations: 6MWDT, 6-minute walk distance test; ACEI, angiotensin-converting enzyme inhibitors; ARNi, angiotensin receptor neprilysin inhibitors; CABG, coronary artery bypass grafting; ICD, implantable cardioverter-defibrillator; _e_GFR, estimated glomerular filtration rate; QTc, corrected QT interval; LV, left ventricular; Pts, patients; SGLT2i, sodium glucose co-transporter 2 inhibitors; _S_VT, sustained ventricular tachycardia; M, mean; Me, median.

### 3.2 Mitochondrial DNA Genotypes

Comparison of the mtDNA polymorphisms abundance between the groups revealed that patients with _S_VT less frequently had mtDNA A2706G substitution (55.7% vs. 76.9%, *p* = 0.015). The other two polymorphisms G3010A and G9055A did not show statistically significant differences. A detailed comparison of mitochondrial nucleotide transition assessment parameters between groups is shown in Table 2.

**Table 2.** **Mitochondrial nucleotide transition assessment parameters**.

	mtDNA A2706G, n (%)		mtDNA G3010A, n (%)		mtDNA G9055A, n (%)	
	A	G	G	A	G	A
1st group Pts with _S_VT (n = 70)	31 (44.3)	39 (55.7)	13 (18.6)	57 (81.4)	7 (10)	63 (90.0)
2nd group Pts without _S_VT (n = 52)	12 (23.1)	40 (76.9)	12 (23.1)	40 (76.9)	5 (9.6)	47 (90.4)
*p*-value	0.015		0.542		0.943	

Bold values indicate statistical significance. Abbreviations: A, adenine; G, guanine; Pts, patients; _S_VT, sustained ventricular tachycardia; mtDNA, mitochondrial DNA.

### 3.3 Risk Stratification Analysis of _S_VT

**Fig. 2.**
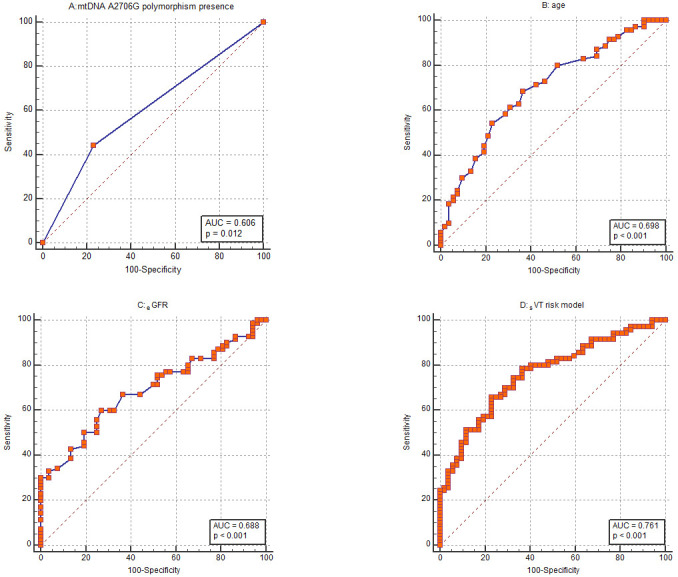
**Receiver operating characteristic (ROC) curves**. ROC curves to assess the ability of (A) mtDNA A2706G polymorphism presence, (B) age, (C) estimated glomerular filtration rate (_e_GFR) and (D) the whole risk stratification model to discriminate patients with and without sustained ventricular tachycardia (_S_VT). mtDNA, mitochondrial DNA; AUC, area under the curve.

Based on the assessed indicators, age was the parameter most strongly correlated with _S_VT. Its discriminative ability was evaluated by receiver operating characteristic (ROC) analysis showing an AUC of 0.698 (95% CI 0.608–0.778) (Fig. 2B). The ability of _e_GFR to distinguish for the _S_VT was analogous with an AUC of 0.688 (95% CI: 0.598–0.769) (Fig. 2C). Concerning the mitochondrial A2706G polymorphism, the ROC curve calculation showed an AUC of 0.606 (95% CI 0.514–0.693) (Fig. 2A).

**Fig. 3.**
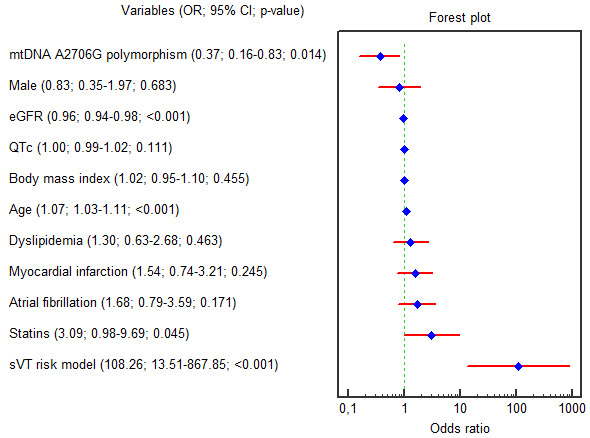
**Forest plot for the univariable logistic regression results**. Abbreviations: 95% CI, 95% confidence interval; _e_GFR, estimated glomerular filtration rate; G, guanine; QTc, corrected QT interval; mtDNA, mitochondrial DNA; OR, odds ratio; _S_VT, sustained ventricular tachycardia.

According to the univariable logistic regression analysis age (OR = 1.073; 95% CI 1.032–1.116; *p* < 0.001), _e_GFR (OR = 0.964; 95% CI 0.943–0.985; *p* < 0.001), absence of mtDNA A2706G polymorphism (OR = 0.377; 95% CI 0.169–0.839; *p* = 0.014) and lipid-lowering treatment (OR = 3.095; 95% CI 0.988–9.692; *p* = 0.045) were independently associated with the _S_VT (Fig. 3).

Only three variables (age, _e_GFR and absence of mtDNA A2706G substitution) remained considerable in multivariable regression analysis, even after amendment for male gender, QTc and presence of MI in anamnesis (OR = 1.055, 95% CI 1.009–1.103, *p* = 0.017; OR = 0.974, 95% CI 0.949–0.999, *p* = 0.041; OR = 0.335, 95% CI 0.141–0.797, *p* = 0.013; respectively). The beta coefficients in the logistic equation match the OR natural logarithm (ln(OR) = β; OR = e^β^).

### 3.4 Risk Score Assessment for _S_VT Development

A risk score for _S_VT was established using logistic regression according to collected data. Three variables (age, _e_GFR and absence of mtDNA A2706G polymorphism) were added in the final model syne these indicators remained significant in multivariable logistic regression, even after amendment for male gender, QTc and presence of MI in anamnesis. The AUC was calculated to assess the discriminatory potential of the risk stratification score. The risk model showed the ability to discriminate evidenced by an AUC of 0.761 (Fig. 2D). At a cut-off point of >0.6; the model demonstrates a sensitivity of 65.71% and a specificity of 76.92% in discriminating against patients with ICD implantation indications regarding _S_VT. Our risk stratification model showed a positive prognostic value of 80.00% and a negative prognostic value of 53.85%.

The age and _e_GFR values were inserted in the score equation (in year and mL/min/1.73 m^2^, respectively). The substitution of adenine to guanine in mtDNA A2706G was indicated in the score equation by assigning a value of 1 (apparent) or 0 (in-apparent). The result of the logistic equation below is the probability (p) of _S_VT development. If the score is >0.6, this risk score allows identification of individuals with ICD implantation indications at an increased risk for _S_VT and following intensified therapy.

Eqn. 1: Probability (p) of _S_VT development.

$$\begin{array}{c} p=\frac{1}{1+e^{-z}}\\ z=-0.52-1.09\times \text{mtDNA A2706G presence }+0.05\times \text{ age }\,\left[\text{year}\right]-0.02\times {}_{\text{e}}\text{GFR}\,\left[\mathrm{mL}/\min/1.73\;\,m^{2}\right] \end{array}$$

## 4. Discussion

As far as we know, the current results provide the first argument of an association between _S_VT with an mtDNA A2706G polymorphism in patients with ICD implantation indications. In this study, we found that the absence of an mtDNA A2706G substitution was associated with the risk of _S_VT development. Besides, the correlation between the absence of an mtDNA A2706G substitution and _S_VT remained after adjusting for conventional cardiovascular disease risk factors.

The adenine in position 2706 is a distinguishing marker for mitochondrial haplogroup H which is the most frequent mtDNA haplogroup in European populations. It should be noted that the human mtDNA reference sequence differs from the ancestral sequence and belongs to the haplogroup H, and the protective effect of “absence of the A2706G polymorphism” should be considered as a risk effect of the haplogroup H. This position is located in the mitochondrial gene that encodes *16S rRNA* for mitochondrial ribosomes. It might be possible that this polymorphism can somehow influence *16S rRNA* structure, and therefore, efficiency of mitochondrial translation. In addition, this position is situated at the end of the gene for the mitochondrial peptide humanin which is known as a signal peptide with cardioprotective and neuroprotective effects [15]. The G3010A polymorphism is also located in the mitochondrial *16S rRNA* gene and can be found in different mtDNA haplogroups. The G9055A polymorphism is located in *MT-ATP6* (mitochondrially encoded adenosine triphosphate synthase 6) gene, leading to the amino acid substitution *Ala177Thr* in the ATP synthase subunit A. Several studies were published that suggested a role of the haplogroup H in the risk of cardiovascular diseases, including ischaemic cardiomyopathy [16], hypertrophic cardiomyopathy [17], dilated cardiomyopathy [18], ischemic heart disease and myocardial infarction [19, 20]. Our results correspond to this common tendency of cardiovascular risk effect of haplogroup H (i.e., absence of A2706G substitution), revealing the same allele risk effect for _S_VT in our patients.

Dysfunction of the mitochondria has been associated with a wide range of cardiovascular pathologies such as HF, cardiomyopathy and arrhythmias [4, 21]. However, most of the evidence was obtained in patients with hereditary diseases caused by genetically confirmed mtDNA abnormalities with strong effects, and the clinical involvement of mtDNA variations in the population as a whole is underinvestigated.

The exact mechanism underlying the association between mtDNA and VTA is unclear. Cardiomyocytes are cells with a high energy demand using mitochondrial respiration and energy production to maintain electrical activity and contractility. It is estimated that approximately 30% of the cardiac ATP generated by mitochondria is used for sarcoplasmic and sarcolemmal reticulum ion channels and transporters, which are required for the cardiomyocytes electrical activity [5, 22]. It has been shown that mtDNA changes in peripheral blood cells correlated with that of cardiomyocytes, suggesting that mtDNA in PBMC may serve as a marker for mitochondrial function in the heart [23]. Accordingly, it is possible that mtDNA changes detected in PBMC could contribute to mitochondrial dysfunction in cardiac cells compromising ATP production and energy supply to ion channels and transporters, leading to altered ion homeostasis, membrane excitability, and cardiac arrhythmias [5]. In our study we did not study the association between SCD and mtDNA polymorphisms. But, according to our results patients with _S_VT less frequently had A2706G a lower mtDNA polymorphism frequency (55.7% vs. 76.9%, *p* = 0.015). Also, the multivariable logistic regression analysis demonstrated that the absence of the A2706G mtDNA polymorphism (OR = 0.335, 95% CI 0.141–0.797, *p* = 0.013) was independently associated with the _S_VT even after adjustment for male gender, QTc and history of MI. The mtDNA haplogroup H, one of the marker polymorphisms of which is A2706G, is widely represented in Western Eurasia, where its share is about 40% [24]. In this regard, it is impossible to confirm unambiguously whether a described polymorphism A2706G has an effect on the development of VT in patients or the entire complex of substitutions inherent in this haplogroup. In our study, the identification of this substitution made it possible to recognize patients who are carriers of Haplogroup H. Therefore, it would be more correct to define that patients who are carriers of haplogroup H, determined by the carriage of the polymorphic variant A2706G, have a higher risk of VT than non-carriers. Otherwise, to determine the entire mtDNA sequence of each patient, it would be necessary to conduct whole genome sequencing, which is unjustified.

Old age and decreased _e_GFR are well-known risk factors for VTA [25, 26, 27]. In the Weidner K *et al*. [25] study middle-aged patients (40–60 years old) were compared to older ones (>60 years old). Amongst 2259 of the included patients, VTA was more frequent in the >60 year old group (50% vs. 59%, *p* = 0.001) [25]. Elderly patients were more often associated with all-cause mortality at 2.5 years (27% vs. 50%; hazard ratio (HR) = 2.137; 95% CI 1.809–2.523; *p* = 0.001) and the secondary endpoints [25]. The results of research by Weidner K *et al*. [25] suggest that increasing age is associated with increased mortality in VTA patients. In our study patients with _S_VT were significantly older than patients without _S_VT (66.9 ± 9.9 year vs. 59.5 ± 10.6 year, *p* < 0.001). Based on the evaluated parameters, an age of more than 62 years was the indicator most strongly correlated with the _S_VT (AUC 0.698; 95% CI 0.608–0.778; sensitivity 68.5%; specificity 63.4%; *p* < 0.001). According to the multivariable logistic regression, age (OR = 1.055; 95% CI 1.009–1.103; *p* = 0.017) was one of the parameters independently associated with the _S_VT.

In a retrospective study by Li *et al*. [27] (n = 503) the decrease in _e_GFR (<60 mL/min/1.73 m^2^) has been demonstrated as an independent risk factor for VTA after acute MI. In our study, patients with acute MI were not included. But, in our cohort, patients with _S_VT had a significantly lower _e_GFR (65.7 ± 19.7 mL/min/1.73 m^2^ vs. 77.9 ± 16.1 mL/min/1.73 m^2^, *p* < 0.001). The cut-off value of _e_GFR associated with _S_VT was 68 mL/min/1.73 m^2^ or less (AUC 0.688; 95% CI 0.598–0.769; sensitivity 60.0%; specificity 73.0%; *p* < 0.001). In accordance with the multivariable logistic regression analysis _e_GFR (OR = 0.974; 95% CI 0.949–0.999; *p* = 0.041) was one of the parameters independently associated with _S_VT.

Previous study focusing on creating a risk model for VTA incidence is limited [28]. Derda A *et al*. [28] showed that in hypertrophic cardiomyopathy patients, the presence of atrial fibrillation, interventricular septum thickness and average peak systolic velocity capable of VTA incidence, were used in prognosing such patients at high-risk for VTA. All three parameters remained significant in multivariate regression (OR = 5.5; 2.1–14.4; *p* < 0.001; OR = 1.09; 1.02–1.17, *p* = 0.014; OR = 0.58; 0.4–0.85; *p* = 0.005, respectively), even after adjustment for age and gender (OR = 6.1; 2.2–16.9, *p* < 0.001; OR = 1.09; 1.01–1.17, *p* = 0.023; OR = 0.59; 0.40–0.86, *p* = 0.006) [28]. The VTA risk model created using these three parameters demonstrated the ability to discriminate evidenced by an AUC of 0.80 [28]. At a cut-off value of >0.39, the model demonstrated a sensitivity of 63% and a specificity of 88% in distinguishing patients with hypertrophic cardiomyopathy regarding the occurrence of VTA [28]. The risk stratification analysis of these patients revealed a positive predictive value of 67% and a negative predictive value of 87% [28]. However, this risk score was more useful in limited cohorts, especially in patients with hypertrophic cardiomyopathy. In our study results, the multivariate logistic regression showed only three independent parameters (age, _e_GFR and the absence of A2706G mtDNA polymorphism) associated with _S_VT. These parameters were added to the ultimate risk score model. The risk score showed the ability to discriminate evidenced by an AUC of 0.761. The developed risk score resulted in an accuracy of 68.9% in correctly predicting _S_VT in our patient sample. In patients with a score above the cut-off value, VT developed more frequently. It is necessary to note that there is no score or model that enables prognosis of 100% probability of the VT. We created a risk score to support physicians to estimate the individual predisposition to VT.

This research has some limitations. One of these is non-randomized and single-center type of study. Study limitations also included the absence of follow-up and relatively small sample size, which may have reduced the significance of the results. Because the study included patients whose ICDs were implanted mostly because of previous potentially lethal ventricular arrhythmias (secondary prevention), one cannot apply current study findings to patients whose ICD was implanted for primary prevention, that was only a small proportion (22%) of the subjects studied. Due to the observational nature of our research, we could identify an association but not establish a causal link between mtDNA and _S_VT. In our study, we researched the carriage of mtDNA polymorphisms of samples derived from the whole blood which may not be the relevant tissue with respect to VT. Future studies with a larger number of primary prevention ICD patients could expand the clinical utility of current study findings.

## 5. Conclusions

In summary, in patients with indications for an ICD implantation, mtDNA polymorphism is associated with _S_VT. Our risk model including age, _e_GFR and the absence of A2706G mtDNA polymorphism may distinguish patients with _S_VT. There are potential directions for expanding the study, such as including larger, prospective, randomized, multi-centered, and diverse patient populations, which would be beneficial to validate our predictive model.

## Availability of Data and Materials

All data reported in this paper will be shared by the lead contact upon request.
